# A Reappraisal of Azhdarchid Pterosaur Functional Morphology and Paleoecology

**DOI:** 10.1371/journal.pone.0002271

**Published:** 2008-05-28

**Authors:** Mark P. Witton, Darren Naish

**Affiliations:** School of Earth and Environmental Sciences, University of Portsmouth, Portsmouth, United Kingdom; Monterey Bay Aquarium Research Institute, United States of America

## Abstract

Azhdarchid pterosaurs were among the most widespread and successful of pterosaur clades, but their paleoecology remains controversial. Morphological features common to all azhdarchids include a long, shallow rostrum; elongate, cylindrical cervical vertebrae that formed a long and unusually inflexible neck; and proportionally short wings with an abbreviated fourth phalanx. While azhdarchids have been imagined as vulture-like scavengers, sediment probers, swimmers, waders, aerial predators, or stork-like generalists, most recent authors have regarded them as skim-feeders, trawling their lower jaws through water during flight and seizing aquatic prey from the water's surface. Although apparently widely accepted, the skim-feeding model lacks critical support from anatomy and functional morphology. Azhdarchids lack the many cranial specialisations exhibited by extant skim-feeding birds, most notably the laterally compressed lower jaw and shock absorbing apparatus required for this feeding style. Well-preserved azhdarchid skulls are rare, but their rostra and lower jaws appear to have been sub-triangular in cross-section, and thus dissimilar to those of skim-feeders and sediment probers. Taphonomic data indicates that azhdarchids predominately inhabited inland settings, and azhdarchid morphology indicates that they were poorly suited for all proposed lifestyles bar wading and terrestrial foraging. However, azhdarchid footprints show that their feet were relatively small, padded and slender, and thus not well suited for wading. We argue that azhdarchids were stork- or ground hornbill-like generalists, foraging in diverse environments for small animals and carrion. Proficient terrestrial abilities and a relatively inflexible neck are in agreement with this interpretation.

## Introduction

Azhdarchids were a highly successful Cretaceous pterosaur clade, distributed virtually worldwide [1] with a fossil record extending from perhaps the Aptian-Albian to the end of the Maastrichtian [2], [3]. Azhdarchid fossils are most abundant in the Upper Cretaceous and are best known for gigantic forms like *Quetzalcoatlus northropi* from the Maastrichtian of the USA (wingspan c. 10 m). Not all azhdarchids are enormous however, and the clade also includes smaller forms such as *Montanazhdarcho minor* from the Campanian of Montana (wingspan c. 2.5 m), *Zhejiangopterus linhaiensis* from the early Campanian of China (wingspan c. 3.5 m), and *Bakonydraco galaczi* from the Santonian of Hungary (wingspan c. 3.5 m) [4]–[6]. Some taxa apparently exceeded *Q. northropi* in size, with *Hatzegopteryx thambema* from the Maastrichtian of Romania having an estimated wingspan greater than 12 m [7]. Earlier remains of azhdarchids currently await re-evaluation: a possible Upper Jurassic azhdarchid from Tanzania has recently been suggested to be a ctenochamatoid [8], while the the Jiufotang Formation taxon *Eoazhdarcho liaoxiensis*, once thought to be a diminutive azhdarchid with a wingspan of 1.6 m [3], has since been suggested to represent a basal azhdarchoid [9] or pteranodontid [10].

**Figure 1:**
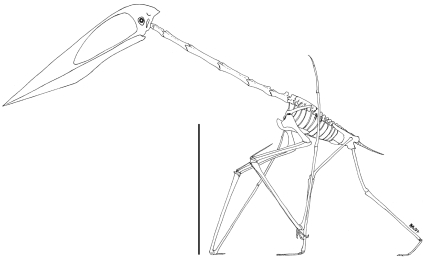
Reconstructed skeleton of *Zhejiangopterus linhaiensis* based on [40] and [47]. Scale bar represents 500 mm.

Azhdarchids share a suite of characters with a number of smaller Cretaceous pterosaurs (the tapejarids from South America and China, and the tupuxuarids from South and possibly North America), and are united with them in the pterodactyloid clade Azhdarchoidea [11]–[13]. Azhdarchid fossils are relatively abundant compared to those of other azhdarchoids: while many are fragmentary, complete skeletons are known, but are yet to be adequately described. Despite these problems, we recognize that, while some minor morphological differences in skull and limb proportions can be recognised between azhdarchid genera (e.g. *Quetzalcoatlus*, *Zhejiangopterus*, *Hatzegopteryx*), broad morphological similarities can be identified across the group: all azhdarchids exhibit large skulls with long, edentulous rostra, elongate, cylindrical cervical vertebrae, proportionally short wings with an abbreviated fourth phalanx, and elongate hindlimbs [12]. These anatomical features, combined with the large size of some taxa, make azhdarchids one of the most striking and distinctive pterosaur groups (Figure 1).

By far the greatest controversy in azhdarchid studies concerns their paleoecology, the resolution of which has remained enigmatic due to their unusual anatomy, the sedimentary settings in which their fossils are found, and a general lack of research into pterosaur biomechanics other than those concerned with flight (e.g. [14]–[16]). Many authors have made speculations about azhdarchid paleoecology [2], [17]–[32], often showing a preference for a skim-feeding lifestyle. The only extant obligate skim-feeders are the skimmers, *Rynchops* (Laridae, Charadriiformes), a highly specialised group of gull-like birds that fly low over the water surface, trawling their unusually elongate, laterally compressed lower jaws through the water and grabbing animal prey that they contact. In contrast to that of many other birds, the feeding behaviour and cranial morphology of skimmers has been well described (e.g. [33]–[36]). Two tern species, the Royal tern *Thalasseus maximus* and Caspian tern *Hydroprogne caspia*, are capable of facultative skim-feeding but lack the many unusual features of *Rynchops* [37]. Despite the preference many pterosaur workers have for the concept of skim-feeding in azhdarchids, little work on the biological feasibility of this, or other, feeding strategies have been performed. While most azhdarchids are known from isolated fragments, the complete skeletons that are known provide us with enough information to assess paleoecological hypotheses for the group.

### Proposed Azhdarchid Lifestyles

Pterosaurs as a whole are most often thought to have been shorebird-like piscivores, with some imagined as insectivores or durophagores [24]. Azhdarchid anatomy has been difficult to interpret (as evidenced by [38], [39]), and it was not until relatively complete specimens of the Javelina Formation form *Quetzalcoatlus* were discovered that paleoecological interpretations could be made [2]. The first proposed hypothesis of azhdarchid habits - prompted by the stiff, hyper-elongate neck and large size of *Quetzalcoatlus* combined with the sedimentology of the Javelina Formation - was that *Quetzalcoatlus* was a carrion feeder, using a vulture-like flight style and long neck to probe into dinosaur carcasses [2]. The traditional pterosaur diet of piscivory was ruled out due to the structure of the cervical vertebrae and large size of *Quetzalcoatlus*, and the small size of the streams that would have existed in the environment represented by the Javelina Formation [2].

Langston [17] also suggested that the Javelina paleoenvironment would prevent piscivory, with an absence of fish fossils cited as evidence against a piscivorous lifestyle. Langston's alternative scenario, based predominantly on the association of invertebrate trace fossils with *Quetzalcoatlus*, was that giant azhdarchids fed on burrowing arthropods by probing for them in the substrate [17]. The probing scenario was also favoured by Wellnhofer [24] and Lehman and Langston [26]. Paul [19], [20], Chatterjee and Templin [16] and Witton [32] regarded azhdarchids as terrestrial foragers, suggesting that they patrolled water courses, grabbing fish and other animals. Bennett [31] concluded, based on its relatively robust hindlimb elements, that *Quetzalcoatlus* might have been heron- or stork-like in its ecology, and a heron-like lifestyle was also intimated by Padian [21]. Ősi et al. [6] imagined *Bakonydraco* to have had a diverse diet of both fishes and fruits, with the latter collected whilst the animal walked through sparsely forested environments.

An entirely different paleoecological role was imagined for azhdarchids by Nessov [18]. In describing *Azhdarcho lancicollis* from the Coniacian of Uzbekistan, Nessov [18] suggested that azhdarchids might have skim-fed in the manner of *Rynchops*, writing ‘If it is assumed that the Azhdarchinae could have flown like the Ornithocheirinae and Pteranodontinae – that is, like the Recent skimmers…’ (p. 42). Supposedly, the long neck would enable azhdarchids to reach food at depth whilst flying or swimming, thereby circumventing the need to dive [16], [18]. Mid-air predation of ‘poorly-flying vertebrates’ was also suggested [18].

Of these proposed lifestyles, in-flight piscivory appears to have gained the most acceptance [16], [18], [22], [25], [27]–[30], [40], with skim-feeding being a frequently suggested foraging method [18], [25], [27], [30]. According to these suggestions, the long azhdarchid neck would enable dip- or skim-feeding without damaging the wing-tips [27], [29], whilst the streamlined skull would reduce drag when skim-feeding [27]. Prieto [30] argued that a laterally compressed bill, poor terrestrial abilities, and wing shapes resembling those of highly aerial birds like frigatebirds, swallows and some kites indicated that azhdarchids were specialised for feeding on the wing. Similarities in the gape of *Quetzalcoatlus* and *Rynchops* have been cited as further evidence for skim-feeding behaviour [25].

It is noteworthy that many paleobiological interpretations of azhdarchid anatomy are contradictory. The long, stiff azhdarchid neck is interpreted by some authors as having *impaired* in-flight feeding [2], [6], [17], [32] but is taken to *suggest* in-flight feeding by others (e.g. [18], [25], [27], [30]). Similarly, the azhdarchid wing planform is evidence of a slow, soaring flight to some [2] but indicative of fast, dynamic flight to others [16], [30]. Such contradictions highlight the lack of research into azhdarchid functional morphology, a situation further hampered by inadequate descriptions of azhdarchid fossils. It is also notable that, rather than being based on details of azhdarchids themselves, some of these hypotheses rely strongly on comparisons with other pterosaurs: when proposing the azhdarchid skimming hypothesis, Nessov [18] directly compared *Azhdarcho* with ‘Ornithocheirinae’ and ‘Pteranodontidae’ (see quotation, above), and Kellner and Langston regarded skim-feeding as plausible for *Quetzalcoatlus* on the basis that it had been ‘previously advocated for *Rhamphorhynchus* … and later assumed for many other pterosaurs, including the larger toothless pterosaurs’ ([25] p. 231). Given that the lifestyles of other pterosaurs are no more extensively researched than those of azhdarchids, this type of argumentation is weak and ignores the many obvious anatomical distinctions between these pterosaur taxa. Oversimplified views that all pterosaurs were ecologically alike have undoubtedly added to the controversy surrounding azhdarchid feeding methods.

## Methods

We have assessed the likelihood of proposed azhdarchid lifestyles through both a functional analysis of the azhdarchid skeleton, and by employing comparisons with the anatomy of modern animals occupying similar niches to those inferred for azhdarchids. We acknowledge that azhdarchids may have possessed unique adaptations to particular lifestyles that are not seen in any modern taxa, but pterosaur anatomy is not so disparate from that of extant animals that some functional convergence should not be expected through adaptation to a similar lifestyle (e.g. large, weight-spreading feet for wading, robust jaw joints for skim feeders). An assessment of azhdarchid trace fossils was used to evaluate their terrestrial competence. Additionally, we tested the notion that azhdarchids were, as has been suggested for other pterosaurs, predominately shore-dwelling animals with a quantitative assessment of their geological context. Using sedimentary and other fossil remains as evidence of paleoenvironment, azhdarchid fossils were scored as occurring in settings that were fully terrestrial, coastal, marine with terrestrial input or fully marine with no terrestrial input. This dataset of 33 azhdarchid-bearing localities was then assessed along with consideration of the completeness and abundance of their remains to develop an understanding of preferred azhdarchid habitat.

## Results

### Inferences from the azhdarchid fossil record

**Figure 2:**
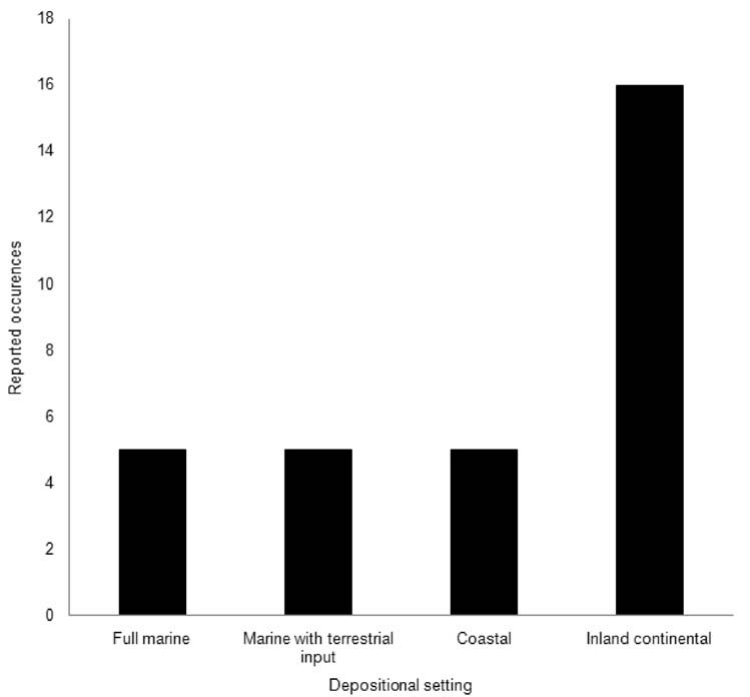
The terrestrial skew of azhdarchid fossils based on data in Table 1.

The notion that pterosaurs were predominantly analogues of seabirds seems to have arisen from their frequent occurrences in marine deposits [24], and this inference has been applied to azhdarchids by several authors [18], [25], [41], [42]. However, increasing numbers of pterosaurs are being recovered from inland deposits (e.g. [43]–[45]), implying that the supposed connection pterosaurs had to marine environments may reflect artefacts of preservation rather than actual habitat preference. Notably, most azhdarchids are found in continental fluvial deposits [44], [46], a condition perhaps best demonstrated by the occurrence of *Quetzalcoatlus* 400 km from the nearest contemporary shoreline [2]. At least 16 azhdarchid occurrences (52% of surveyed material) are from inland sediments (e.g., fluvial or alluvial sediments, overbank deposits: Table 1, Figure 2). Furthermore, all but five marine or coastal occurrences are associated with terrestrially-derived fossils such as non-avian dinosaurs, plants and amphibians (83% of surveyed literature: Table 1, Figure 2), and only terrestrial deposits preserve remains of associated azhdarchid individuals [2], [6], [40], [43], [47]. Moreover, the most complete, best preserved azhdarchid fossils are found in terrestrial settings, whereas fossils found in marine settings are generally isolated bones or bone fragments. Possible azhdarchid footprints are also only known from inland lacustrine settings [46].

**Table 1:** Geological context of azhdarchid fossils.

Taxon	Locality	Lithology	Associated fossils	Depositional setting	References
*Aralazhdarcho*	Shakh-Shahk locality, Kazakhstan	Red beds	Amphibians, dinosaurs, crocodiles, mammals	Continental	[52], [42]
*Arambourgiania*	Balqa Group, Jordan	Phosphates	Fishes, turtles, marine reptiles, crocodiles, dinosaurs	Marine	[58]
*Azhdarcho*	Taykarshinskaya unit, Uzbekistan	Lenticular sands	Turtles, amphibians,	Coastal	[18]
cf. *Azhdarcho*	Laño Locality, Basque Country	Sands and clays	Fish, amphibians, reptiles and mammals	Continental	[121]
*Bakonydraco*	Csehbánya Formation, Hungary	Silts and sandstones	Turtles, amphibians, lizards, dinosaurs, crocodiles, fishes	Continental; fluvial	[6]
*Hatzegopteryx*	Densuş-Ciula Formation, Romania	Siltstone	Dinosaur eggs, amphibians, turtles, crocodiles, mammals	Continental; fluvial	[7]
*Montanazhdarcho*	Two Medicine Formation, USA	Oxidised, cross bedded sands	Dinosaurs, chelonians, freshwater molluscs, lizards	Continental; lacustrine	[122], [123]
*Phosphatodraco*	Oulad Abdou Basin, Morocco	Phosphates, marls	Mosasaurs, sauropterygians, turtles, fish, sharks	Marine	[124]
*Quetzalcoatlus*	Javelina Formation, USA	Siltstone	Dinosaurs, plants	Continental; fluvial	[2]
*Quetzalcoatlus*(?)	Oldman Formation, Canada	Grey silt/white sandstone	Dinosaurs, plants, turtles, crocodiles	Continental; fluvial	[125]
cf. *Quetzalcoatlus*	Marnes d'Auzas Formation, France	Coarse sandstone	Turtles, crocodiles, dinosaurs	Freshwater-brackish	[44]
cf. *Quetzalcoatlus*	Hell Creek Formation, USA	Cross bedded sands	Angiosperm leaves	Continental; fluvial	[60]
*Zhejiangopterus*	Tangshang Formation, China	Volcanic tuffs	Dromaeosaur	Continental; lacustrine	[4]
Azhdarchidae indet.	Azizbek District, Armenia	Lenticular sands	Ammonites, leaf imprint	Marine*	[41]
Azhdarchidae indet.	Pudovkino Formation, Russia	Phosphates	Echinoderms, oysters, belemnites, mosasaurs	Marine	[1]
Azhdarchidae indet.	Volga Region, Russia	Phosphates	Plesiosaurs, mosasaurs	Marine	[126]
Azhdarchidae indet.	Miria Formation, Australia	Chalk	Marine invertebrates, saurichian humerus(?), reptilia indet.	Marine	[127]
Azhdarchidae indet.	Saint Foy site, France	‘Non-marine sediments’	Dinosaurs, crocodiles	Continental	[128]
Azhdarchidae indet.	Montplasir site, France	Red and purple marls	Charophytes, dinosaurs, crocodiles, chelonians	Continental*	[129]
Azhdarchidae indet.	Sierra Perenchiza Formation, Spain	Mottled siltstone	Fishes, crocodiles, dinosaurs, frogs	Continental; lacustrine	[117]
Azhdarchidae indet.	Dinosaur Park Formation, Canada	Fine/medium sands	Dinosaurs, amphibians, mammals, chelonians	Continental; fluvial	[130]
Azhdarchidae indet.	Mifune Group, Japan	Coarse sands, muddy lenses	Dinosaurs, crocodiles, turtles, fishes, mammals	Continental	[118]
Azhdarchidae indet.	Hakobuchi Group, Japan	Sandstones, mudstones, lignites	Marine invertebrates, mosasaurs, cehlonians	Coastal	[28]
Azhdarchidae indet.	Ksar es Souk, Morocco	Coarse sands	Dinosaurs, fish, crocodiles, chelonians	Continental	[131]
Azhdarchidae indet.	Paki, Sénégal	Quartzic sandstones	Molluscs, echinoderms, angiosperms	Coastal	[132]
Azhdarchidae indet.	Glen Rose Formation, USA	Micrite	Logs, plant cuticles, ostracodes, fish	Marine	[133]
Azhdarchidae indet.	Kita-ama Formation, Japan	Coarse sandstone	Ammonites, inoceramid bivalves, chelonians, dinosaurs	Marine	[134]
Azhdarchidae indet.	Seidan Formation, Japan	Siltstone	Gastropods	Marine	[135]
Azhdarchidae indet.	Peedee Formation, USA	Alternating limestones and sands	Mardine invertebrates, marine reptiles, dinosaurs	Marine	[136]
Azhdarchidae indet.	Portezuelo Formation, Argentina	Siltstones, sandstones	Dinosaurs, crocodiles	Continental	[137]
?Azhdarchidae	Kem Kem Region, Morocco	Red beds	Fish, turtles, lizards, crocodiles, dinosaurs, pterosaurs	Coastal	[53]
?Azhdarchidae	Illd Formation, Japan	Shales	Bivalves	Marine	[138]

Depositional settings with asterisks indicate paleoenvironmental interpretations by authors based on associated fossils and sedimentology.

Although interpreting the ecology of extinct organisms from their depositional settings is problematic due to taphonomic influences [48], [49], a significant body of evidence indicates that azhdarchids were denizens of continental settings. Their relative abundance in terrestrial settings contradicts the expectation that marine settings are more conducive to fossilisation than continental environments, and we suggest that this reflects a genuine signal of higher azhdarchid populations inland [50]. This is supported by the relative concentration, completeness and articulation of continentally-preserved azhdarchids compared to their marine counterparts. While scavenging and decay are important taphonomic factors in both marine and continental settings [51], it is significant that marine azhdarchid remains are typically isolated limb bones or cervical vertebrae, as these are among the most readily transported skeletal components in modern vertebrates (Voorhies Groups 1 and 2 of [49] and references therein). Additionally, the inclusion of allochthonous terrestrial material in many azhdarchid-bearing marine horizons implies that the azhdarchid material preserved in these units could also be derived from terrestrial settings. These lines of evidence converge to suggest a strong continental bias for azhdarchid fossils which is most parsimoniously interpreted as an indication of preferred habitat.

An alternative hypothesis suggests that azhdarchids were migratory [16], [25], [41] and that their occurrences inland may result from deaths that occurred *en route* to other locations [25]. While the volant abilities of azhdarchids would certainly permit migratory behaviour, this hypothesis is speculative and, moreover, it is highly unlikely that the vast majority of azhdarchid fossils became associated with continental deposits through chance deaths of migrating animals. Moreover, this hypothesis requires an explanation as to why there are relatively few azhdarchids from coastal or marine deposits. It is far more parsimonious to interpret the overwhelming number of azhdarchid occurrences in terrestrial settings as representing the favoured habitat of these animals. Indeed, strong evidence that azhdarchids inhabited continental environments also comes from their anatomy: numerous details of the azhdarchid skeleton suggest greater terrestrial competence than that of many other pterosaurs.

### Functional anatomy

#### Skull

**Figure 3:**
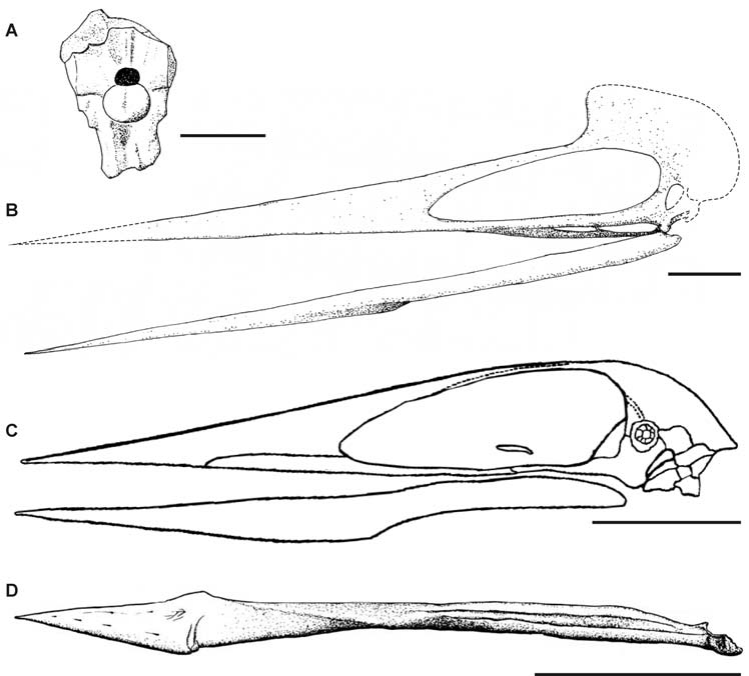
Azhdarchid skull material. A, occipital region of *Hatzegopteryx* (modified from [7]), B, reconstruction of *Quetzalcoatlus* sp. based on photographs in [25]; C, *Zhejiangoperus* (modified from [40]); D, mandible of *Bakonydraco* (modified from [6]); Scale bars represent 100 mm.

Azhdarchid skull material is rare, with complete or near-complete skulls only known for *Quetzalcoatlus* sp. and *Zhejiangopterus linhaiensis* (Figure 3B–C; [25], [40]). Unfortunately, these specimens have suffered crushing that obscures many details bar those of the skull profiles. Fragmentary but three-dimensional skull material is known, however, for *Azhdarcho lancicollis* [18], *Hatzegopteryx thambema* (Figure 3A; [7]), *Bakonydraco galaczi* (Figure 3D; [6]) and *Aralazhdarcho bostobensis* [42], [52]. An incomplete three-dimensional rostrum, probably from an azhdarchid, is also known from the Cretaceous of Morocco [53].

The azhdarchid skull is long, lightly built and approximately triangular in lateral profile (Figure 3; [25], [40]). Azhdarchids appear to have variable jaw width-length ratios, ranging from 0.12 in *Quetzalcoatlus* sp. to 0.22 in *Bakonydraco galaczi*. The giant azhdarchid *Hatzegopteryx* appears to have a particularly wide skull (500 mm across the quadrates), but the relationship between this and skull length cannot be ascertained due to its fragmentary nature. Azhdarchid mandibles are slender in lateral view with a mandibular symphysis that extends for 45–66% the length of the jawline. The jaws are straight and edentulous [6], [18], [25], [40], although *Bakonydraco* possesses a low transverse ridge across the dorsal surface of the mandibular symphysis [6]. The occlusal surfaces of the jaws are flat save for low ridges along the jaw margins [6], [18], [25]. *Quetzalcoatlus* may have a ventrally convex palatal region beneath the posterior half of the nasoantorbital fenestra: four *Quetzalcoatlus* specimens with this region preserved have broken palatal elements that extend below the ventral margin of the maxilla and jugal bars (Figure 3B; [25]), suggesting that their palates were similar to those of the tupuxuarid azhdarchoid *Tupuxuara* [54]. The azhdarchid mandibular joint is typical of pterosaurs in being a simple hinge permitting slight lateral movement of the mandible during jaw extension. A process on the glenoid fossa of *Quetzalcoatlus* may have prevented lateral movement of the quadrates when the jaws were at maximum gape [25]. The cranium is relatively small with a ventrally oriented occiput set within a sculpted occipital region [7], [12]. The occipital condyle is only known in three-dimensions from *Hatzegopteryx*: it reveals a large, well developed hemispherical condyle with no obvious ‘neck’ [7].

Several lines of evidence suggest that azhdarchid jaw musculature was relatively weak. Attachment sites for jaw musculature on the mandible appear to be small: the mandibular rami show no dorsoventral expansion around the coronoid region, and the retroarticular process is no longer than 3% of the jaw length [6], [25], [40], indicating the small sizes of M. pterygoideus posterior, M. adductor mandibulae posterior and M. intramandibularis (assuming a jaw myology similar to that of extant archosaurs – see [55]). Similarly, the supratemporal fenestrae are reduced and positioned at a low angle to the palate in *Zhejiangopterus* [40], providing the already small M. pseudotemporalis with relatively little mechanical advantage during jaw adduction. The large subtemporal fenestra of *Quetzalcoatlus* and *Hatzegopteryx* indicates the most important jaw adductor was M. pterygoideus anterior, occupying approximately 8% of the jaw length in *Quetzalcoatlus* and 15% of the estimated jaw width in *Hatzegopteryx*. This simplified jaw myology is similar to that of birds in which M. pterygoideus anterior is also the dominant adductor [56] and would have provided azhdarchids with a relatively weak bite. Furthermore, the elongate azhdarchid rostrum would have resulted in low bite pressures delivered at the jaw tips and the straight jaw margins would provide no concentration of bite force. We therefore conclude that azhdarchid jaws were ill suited for demanding feeding techniques or for subduing large, struggling prey, and were better adapted for handling relatively small or immobile food items.

#### Cervical vertebrae

**Figure 4:**
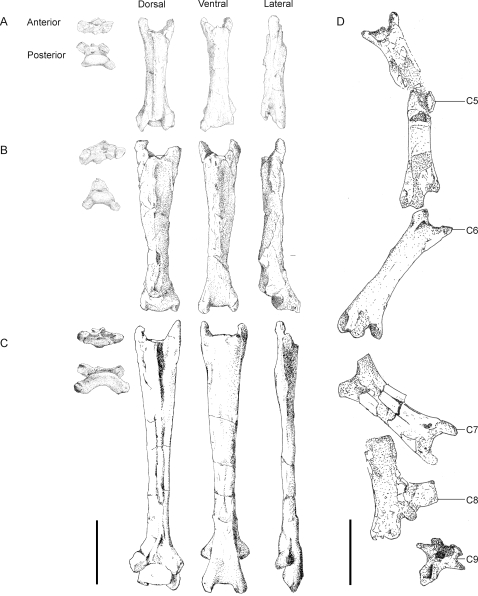
Azhdarchid cervical vertebrae. A–C, *Quetzalcoatlus* cervical vertebrae 3–5; D, *Phosphatodraco* cervical series *in situ* (after [47]). Scale bars represent 100 mm.

Cervical vertebrae are perhaps the best known elements of azhdarchid anatomy, with detailed descriptions provided by Howse [57], Frey and Martill [58], Martill et al. [29], Pereda Suberbiola et al. [47], Godfrey and Currie [59] and Henderson and Peterson [60]. They are among the most common of azhdarchid remains, although complete cervical series are only known for *Quetzalcoatlus* sp., *Zhejiangopterus* and *Phosphatodraco* (Figure 4; [40], [47], [57]). The vertebrae are typically elongate and strongly procoelous with low, ridge-like neural spines and no transverse processes (but see below), although the neural spines are somewhat more developed on the anterior vertebrae. Vestigial cervical ribs occur in small, possibly immature individuals and are entirely fused to the ventral surfaces of the prezygapophyses, with the sutures between ribs and vertebrae absent in larger individuals [59]. Azhdarchid cervicals typically possess large zygapophyses and exapophyses that extend at low angles relative to the vertebral long axis [29], [57], [47]. The postzygapophyses have posteroventrally oriented articular surfaces, with opposing faces seen on the prezygapophyses. The centra extend posteriorly beyond the postzygapophyses but terminate well before the anterior extremity of the prezygapophyses [57]. The condyle is prominent and shows anterodorsal articular surfaces. The cotyle is dorsoventrally asymmetrical, being deeper and broader in the dorsal half. A posteroventrally projecting hypapophysis [59], [60] extends from the cotyle to insert between the exapophyses of the following vertebra. Transverse sections taken at mid-vertebral-length show sub-circular or slightly dorsoventrally flattened cross sections, but both cotyles and condyles are dorsoventrally compressed [29], [57], [60]. The *Phosphatodraco* cervical series suggests that cervicals eight and nine are exceptions to the slender construction present in the rest of the cervical series, showing relatively large neural spines and, on cervical nine, prominent transverse processes (Figure 4D; [47]). Larger neural spines have also been reported on cervical eight of *Quetzalcoatlus* and *Zhejiangopterus* [8]. The postzygapophyses of cervical eight also project at higher angles relative to the long axis of the vertebrae, but the condition of the prezygapophyses is not known [47].

The crushed or fragmentary nature of most azhdarchid vertebrae has prevented accurate interpretation of their mechanics (e.g. [2], [24], [29], [58]), and, although well preserved azhdarchid cervicals have recently been described [47], [59] qualifying the degrees of articulation between vertebrae remains difficult. It appears that ventroflexion of the neck was limited by the presence of prominent exapophyses and hypapophyses [29], with flexibility decreasing posteriorly (Unwin, pers. comm. 2008). Rotation and lateral flex was hampered by the dorsoventrally compressed centra, laterally flared prezygapophyses and interlocking exapophyses and hypapophysis. Articulation was particularly restricted in the mid-cervicals where the zygapophses are longer and broader, and the absence of posteroventral articulatory faces between these vertebrae seems to confirm their limited ventroflexion.

By contrast, the condylar articular surfaces suggest that a moderate amount of dorsal extension was permitted between vertebrae. The bulbous posterodorsal convexity of the condyle would have facilitated moderate degrees of dorsal rotation during which the prezygapophyses would slide ventrolaterally beneath the postzygapophyses of the preceding vertebra (a motion confirmed by the articular surfaces of these processes). The dorsoventrally compressed but laterally expanded condyle and cotyle appear to have been hinge-like in that they permit flexion in one plane (sagittal) to the exclusion of others. The longer prezygapophyses of the mid-cervicals may have limited extension in this region, but the anterior-most vertebrae have shorter zygapophyses that may have permitted greater degrees of dorsal rotation. These observations are corroborated by comparisons of zygapophyseal shape with animals bearing flexible necks: forms with increased cervical flexibility have short, high-angle zygapophyses with mediodorsally inclined articulator facets, allowing the zygapophyses to slide as the neck flexes. Manipulation of the posterior *Azhdarcho* and *Quetzalcoatlus* cervical series suggests that very little extension was permitted at the neck base (Unwin, pers. comm. 2008).

The relative inflexibility of the mid-cervicals may have served to strengthen inter-vertebral joints, thereby forgoing the need for large epaxial ligaments. The reduced extent of such soft tissue is verified by the lack of sculpting for deep muscle or ligament attachment on all azhdarchid cervicals with the exception of cervicals eight and nine, which contrast with their predecessors in bearing prominently developed neural spines and, on cervical nine, transverse processes [47]. The prominent processes of cervical nine indicate it is probably a ‘dorsalised’ cervical akin to that seen in *Pteranodon* [31], leaving cervical eight to form the posterior-most ‘neck element’ of the cervical series. The relatively sculpted morphology of these vertebrae indicates that they anchored larger epaxial muscles than the preceding cervicals that were probably integral in controlling elevation of the neck. Additional anchorage for neck-elevating tissues would be located on the notarium, scapulacoracoid and sternum (as partially reconstructed for *Anhanguera*; [61]). This is in accordance with observations on modern animals demonstrating that soft tissues at the base of the neck control neck elevation [62].

The atlas-axis articulation may also have had limited flexion, despite proposals that this was a main source of flexibility in the azhdarchid neck [24], [29]. This part of the cervical column is not well known, but the atlas and axis are reported to be fused together and truncated in *Zhejiangopterus* [40] and similar to that of *Pteranodon* in *Quetzalcoatlus* and *Azhdarcho* [31]. Detailed descriptions of this region of the neck have yet to be published, however. A fragmentary *Aralazhdarcho* atlas-axis demonstrates a morphology similar to that of other azhdarchid cervical vertebrae with posterolaterally directed exapophyses and a dorsoventrally compressed condyle [42]. Presumably therefore, this morphology imposed the same limit on rotational and vertical articulation as seen between other cervicals. This observation is supported by the small size of the atlas-axis in *Zhejiangopterus* [40], which appears inadequately sized to anchor powerful skull musculature. The generally inflexible nature of the neck therefore appears to extend from the base of the skull throughout much of the neck.

Combined, these observations suggest a relatively limited range of motion in the azhdarchid cervical series. Azhdarchids probably held their necks in a manner similar to lizards and crocodiles with relatively little curvature of their vertebral columns [63]. Mid-series cervicals were probably held with only minor dorsoflexion, forming a gentle, dorsal arc from the torso. The greater length between the pre- and postzygapophyses compared to the length between cotyle and exapophyses dictates that a slight downturn in the cervical series may have occurred at cervical three. However, the vestigial nature of the neural spines indicates that the nuchal ligament did not play a role in maintaining a cervical ‘S’ shape like that of birds and some mammals [63], or in maintaining the strong dorsal arcs seen in some other pterosaurs [31]. Despite this, the *Hatzegopteryx* supraoccipital bears a well-developed medial ridge similar to that of ornithocheirids [64] and pteranodontids [31] for strong attachment of the nuchal ligament. This medial ridge is not seen in smaller forms such as *Tapejara* [65] or *Rhamphorhynchus* [24], suggesting that the development of a large ligament was an adaptation for the supporting of a heavier neck and head.

#### Wing structure and flight capability

Research into pterosaur flight has principally focused on ornithocheiroids like *Pteranodon* [15], [66]–[69] and *Anhanguera* [70], with some studies investigating multiple pterosaur taxa [16], [71], [72]. Azhdarchid flight has yet to be researched in great detail and some controversy surrounds their flight capability. Paul [73] and Frey et al. [74] concluded that azhdarchids would be able to perform prolonged flapping flight using large flight muscles, with Paul [73] suggesting this for even the largest forms. Other workers have argued that the flight muscles of large pterosaurs were not sufficient to maintain flapping flight [16]–[18], [75] and that they were dynamic soarers akin to modern albatrosses [16], [18]. Similar conclusions were drawn by Lawson [2], but vulture-like static soaring was suggested rather than dynamic soaring.

Drawing conclusions about the flight of pterosaurs is problematic due to our limited understanding of their paleobiology. Modelling flight is particularly difficult for the larger forms due to the lack of equivalently sized extant analogues [72]: the absence of such creates problems in estimating the masses of giant pterosaurs, a critical value in modelling even basic flight attributes such as wing loading and flight speed. Despite many attempts at estimating the masses of giant azhdarchids, little agreement has been achieved. Langston [17] suggested a *Quetzalcoatlus* with an 11–12 m wingspan may have weighed 86 kg and, with a 15.5 m wingspan, 136 kg. Citing Bramwell and Whitfield's [15] aeronautical work with a lightweight *Pteranodon*, Wellnhofer [24] suggested that an 86 kg estimate for a 10 m span *Quetzalcoatlus* could be too high and that its weight may have been comparable to modern ultra-light aircraft. Shipman [76] suggested that an azhdarchid of similar size would have a mass of 126 kg. The results of a multivariate analysis by Atanassov and Strauss [77] gave mass estimates of 90–120 kg depending on body density, but similar techniques used by Templin [78] and Chatterjee and Templin [16] produced estimates of 62–77 kg, a range of figures also cited by Witton [32]. A similar mass of 70 kg was produced by Brower and Veinus [71] using regression analysis of geometrically modelled pterosaurs. Considerably higher estimates are given by Marden [79] and Paul [73] at 200–250 kg, but calculations by Chatterjee and Templin [16] suggest a pterosaur of this magnitude would never become airborne. These calculations are contradicted by Marden [79], and other workers have criticised lower mass estimates for being impossibly low [66], [73], [80].

It is noteworthy that all mass estimates of azhdarchids have been based on methods for which pterosaur soft tissue density has to be estimated. Many pterosaurs exhibited extensive skeletal pneumaticity (e.g. [31], [81]) and azhdarchid vertebrae and humeri were clearly pneumatised [29], [40]. We therefore assume that azhdarchids exhibited pneumaticity in both their soft tissue anatomy as well as in their skeleton. However, given that pneumaticity has been shown to vary considerably among extant birds [82] and has a significant impact on mass estimates [83], we know too little about pterosaur anatomy to accurately predict their masses using density-dependent calculating techniques. A regression analysis of dry skeletal mass relative to total body mass [84], a technique that avoids the complications of estimating body density, generates a mass of 250 kg for a 10 m span azhdarchid, a figure matching the higher mass estimates of Paul [73], [80] and Marden [79]. Moreover, calculating the body volume of a giant azhdarchid suggests that soft tissue densities have to be less than 0.25 g/cm^3^ in order to allow masses of under 125 kg (Witton, unpublished data). We note that the masses of not only giant azhdarchids but all pterosaurs have been grossly underestimated and suggest that the flight calculations based on these hyper-lightweight estimates be treated with caution.

Some conclusions on azhdarchid flight can be drawn from their wing morphology alone. Wing planform is highly diagnostic of flight style in extant animals [85], but controversy over the shape of the pterosaur brachiopatagium has resulted in multiple interpretations of azhdarchid flight style. Langston [17], Wellnhofer [24] and Chatterjee and Templin [16] reconstructed azhdarchids with narrow brachiopatagia extending to the top of the hindlimbs, whereas Frey et al. [74] suggested that the membrane extended to the ankle, forming a much broader wing. No fossilised azhdarchid wing membranes are known, but evidence from anurognathids, campylognathoidids, rhamphorhynchids, ctenochasmatoids and non-azhdarchid azhdarchoids [86]–[91] indicates that ankle-attached wing configurations are more accurate.

**Figure 5:**
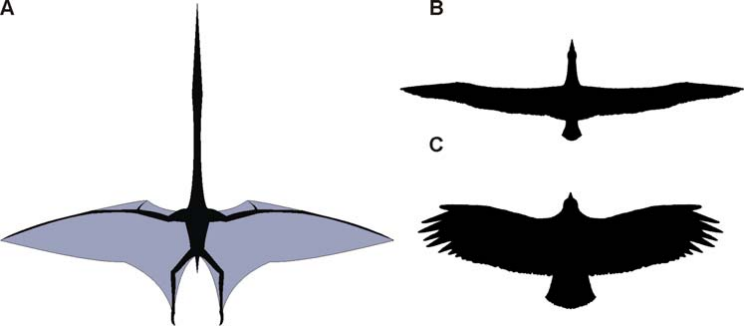
Azhdarchid wing shape. A, reconstructed planform of *Quetzalcoatlus* (wing shape derived from the ‘dark wing’ *Rhamphorhynchus*: see [91]); B, planform of the dynamically soaring wandering albatross (*Diomedea exulans*); C, planform of the statically soaring Andean condor (*Vultur gryphus*). Images not to scale.

Many authors have noted the relatively short wing configuration of azhdarchids, with their abbreviated distal wing phalanges contrasting with their long forearms, the latter a result of hyper-elongate wing metacarpals [12], [16], [17], [74]. These short wings contrast with long hindlimbs, a condition resulting from proportionally elongate femora [12], [17], [74]. Consequently, the forelimb-hindlimb ratio (length of humerus+radius+wing metacarpal/length of femur+tibia) of azhdarchids is low: Chatterjee and Templin [16] suggested a ratio for *Quetzalcoaltus* of 1.13, one of the lowest limb ratios for any pterodactyloid. This contrasts with the limb ratio of 1.45 present in *Zhejiangopterus*: a more typical pterodactyloid limb ratio (unfortunately, a precise ratio for a complete wing to hindlimb length cannot be given as no complete azhdarchid wing fingers have yet been described). Consequently, with their abbreviated wing fingers and long hindlimbs incorporated into the wing membrane, azhdarchids possessed relatively short, broad wings. Reconstructing the azhdarchid wing with broad brachiopatagia (wing shape derived from the ‘dark wing’ *Rhamphorhynchus* [91]) generates an aspect ratio of 8.1 (Figure 5), a value comparable to the aspects of modern storks, raptors and bats that engage in static soaring [85], [92]–[94]. With the relatively low wing loading that such broad wings produce, it is likely that large azhdarchids were also static soarers, using the warmed, rising air of thermals to gain altitude before soaring cross-country. Smaller azhdarchids may have been more capable of complementing gliding with sustained flapping flight than larger forms due to their lower masses and less demanding energetic requirements [16], [72]. These observations are supported by principal component analysis of azhdarchid wing form, with 10 m and 3 m span taxa plotting in the same ecomorphospace as condors, ibises and other thermal soarers [see 85 for further details]. The shape of azhdarchid wings implies that, like modern static soarers, they would have had relatively small turning radii when soaring, but comparatively poor glide performance compared to longer, narrower-winged forms [85], [92]. However, their broad wing area would act in concert with the deep camber produced by the elongate pteroid [40] to generate greater lift than that present in the narrower wing planform of dynamic soarers [70], [92]. This may have been crucial for azhdarchids, allowing them to take off in cluttered inland habitats where wind and topography are too variable to always allow an assisted takeoff. Additionally, it is of obvious benefit to have short, broad wings when taking off in vegetated inland settings [85]. That azhdarchids appear to have wings well adapted for flight in terrestrial environments correlates well with the regular occurrence of azhdarchid fossils in terrestrial strata.

#### Terrestrial capability

**Figure 6:**
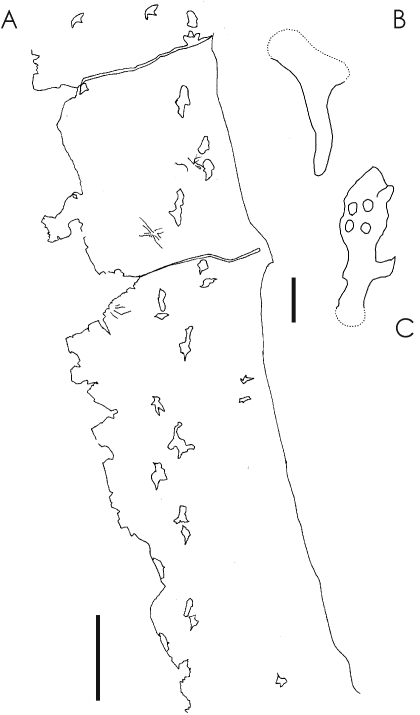
The probable azhdarchid trace fossil *Haenamichnus uhangriensis*. A, the 7.3 m trackway CNUPH.P9; B, *H. uhangriensis* holotype (CNUPH.P2), manus (top) and pes (bottom) prints. Modified from [46]. Scale bars represent 1 m (A) and 100 mm (B).

While the terrestrial abilities of pterosaurs were once regarded as non-existent to poor, reassessments of pterosaur trace fossils indicate that pterodactyloids were competent walkers and runners [23], [95], [96]. Azhdarchid tracks are rare, but large, Upper Cretaceous footprints from Korea (*Haenamichnus uhangriensis*) are thought to have been produced by an azhdarchid (Figure 6; [46]). *Haenamichnus* is distinguished from other pterosaur tracks by its slim shape, rounded heel and apparent absence of large pedal claws, although the manus prints are broadly similar to those of other pterosaur ichnotaxa. A comprehensive comparison between the *Haenamichnus* footprints and azhdarchid feet cannot be made because of the incomplete nature of azhdarchid foot remains [46]: but limited comparisons can be made between the tracks and the partial pes material known from *Zhejiangopterus* [40] and *Quetzalcoatlus* [31]. However, while the identification of *Haenamichnus* as an azhdarchid trace is not proven, it is supported by the Santonian-Campanian age of the tracks, their large size (no other pterosaurs were large enough to produce 35 cm long pes prints), and sub-equal digit lengths (a character shared with *Zhejiangopterus*). Tapejarid feet show some of these characters but bear large claws on all digits [91], while tupuxuarids bear extremely elongate fifth digits. Based on pes print morphology and a Campanian age, tracks referred to *Pteraichnus* sp. from Mexico [97] may also be *Haenamichnus*, albeit produced by a considerably smaller azhdarchid.

Using *Zhejiangopterus* as a template, the *Haenamichnus* trackmaker can be estimated to have stood almost 3 m tall at the shoulder and to have had a wingspan of over 10 m. In concert with the large size of this trackmaker, one *Haenamichnus* trackway has a length of 7 m and is the longest pterosaur trackway yet known [46]. It records a pterosaur moving with an efficient, parasagittal gait [46] rather than in a sprawled posture as suggested by earlier studies (e.g. [98]). In fact, the *Haenamichnus* trackway demonstrates that the presumed azhdarchid trackmaker had a particularly narrow gait with pes prints regularly overlying manus prints, an observation suggesting particularly efficient terrestrial locomotion compared to that inferred from other pterosaur trackways. The *Haenamichnus* pes prints show that the feet possessed soft tissue pads on the digits, metatarsal heads and heel in the manner of some tapejarids [91], [99]) with webbing between the digits [46]. This webbing may also have been present between the digits of the manus [46].

Good descriptions of azhdarchid pes material are lacking, but Bennett [31] reported that the metatarsals of *Quetzalcoatlus* are relatively robust compared to those of *Pteranodon*. This contrasts with the relative pes length of azhdarchids, which was comparatively small for their body size. The pes of *Quetzalcoatlus* is approximately 25% of tibial length, and that of *Zhejiangopterus* 30% tibial length [16], [40]. These figures contrast with the 47% pes-tibial length of *Pteranodon* and *Huaxiapterus* [100], 58% in *Germanodactylus*, 69% in *Pterodactylus* and 84% in *Pterodaustro*. In modern birds, long feet with large surface areas are often associated with swimming or wading behaviour [101], suggesting that the proportionally small feet of azhdarchids were poorly suited for these lifestyles. The hands of azhdarchids are similarly truncated with reduced digits and claws that would function poorly in support on soft substrates. We therefore conclude that azhdarchids were more competent at walking on firm substrates than in marshes, swamps or intertidal environments.

Further evidence of terrestrial competence in azhdarchids stems from their atypically long limbs. With the exception of ornithocheirids with their disproportionately long forearms and small bodies, *Zhejiangopterus* demonstrates longer limbs relative to body length than any other pterosaur. Increased forelimb length is mainly achieved through elongation of the wing metacarpal, creating limb bones proportions similar to those of cursorial ungulates. However, the plantigrade hindlimb does not resemble that of cursorial tetrapods, with a tibia only 20% longer than the femur [40]. Hence, although probably not cursorial, azhdarchids may have been relatively fast, energy efficient terrestrial locomotors merely thanks to the increased stride length allowed by their longer limbs. Further interpretation of long azhdarchid limbs is complicated by the poorly understood selection pressures for long limbs among modern animals [102]–[104], but their potential enhancement of terrestrial proficiency is noteworthy.

## Discussion

### Evaluation of suggested azhdarchid lifestyles

#### Scavenging

Lawson's [2] suggestion that *Quetzalcoatlus* was an obligate scavenger is partially based on the association of sauropod remains with the pterosaur material, but this was refuted by Martill [27] as circumstantial evidence. Some aspects of azhdarchid anatomy appear to support the scavenging hypothesis, particularly their possible adaptations for long-distance static soaring in the manner of vultures [2]. The correlation between high body mass and carcass dominance is well documented in extant scavengers (e.g. [105]–[107]) and the large size of many azhdarchids would almost certainly prove beneficial in this regard, and also permit them to swallow small animal carcasses whole. However, many authors have contrasted the flexible necks of scavenging birds with the stiff necks of azhdarchids and have suggested that the latter would limit any carcass-probing ability [6], [24], [27], [29], [58]. The lack of hooked jaw tips has also been cited as evidence against the scavenging hypothesis [24], [30], but scavenging storks and corvids manage to open carcasses quickly and bite off pieces of flesh without the aid of curved jaw tips [108]. Presumably, the lack of a hooked bill in these forms reflects the fact that scavenging is only part of a broader diet facilitated by a generalized bill morphology [101], [109]. Therefore, it seems almost certain that azhdarchids would have been capable of feeding upon at least some elements of large carcasses, although their long skulls and necks would inhibit their ability to obtain flesh from the deepest recesses of a corpse. However, although carrion was a likely component of azhdarchid diets, they possess no anatomical features to suggest they were obligate scavengers.

#### Probing

**Figure 7:**
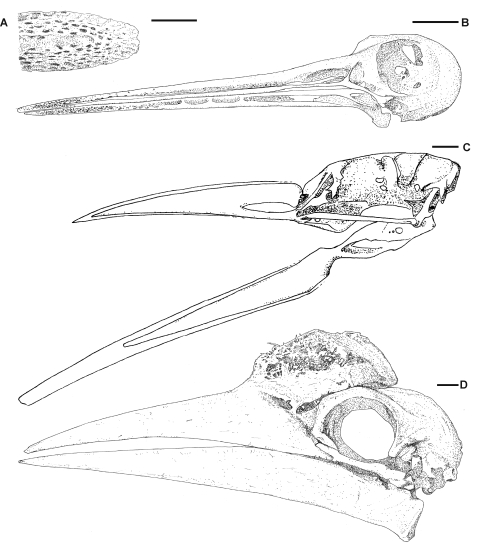
Suggested modern analogues of azhdarchids. A, anterior premaxilla of the western sandpiper (*Calidris mauri*) showing densely packed Herbst corpuscles, dorsal view [after 110]; B, skull of the probing common snipe (*Gallinago gallinago*); C, skull of the black skimmer (*Rynchops nigra*) ; D, skull of the northern ground hornbill *(Bucorvus abyssinicus*). Scale bars represent 1 mm (A) and 10 mm (B–D).

The suggestion that azhdarchids may have probed into sediments in search of infaunal invertebrates is based on the association of *Quetzalcoatlus* remains with invertebrate trace fossils [17], [24], [26], but this evidence is also circumstantial [27], [29]. The restricted ventral flexion of the azhdarchid neck is problematic for this hypothesis [27], [29], as is the cross-sectional shape of the rostrum [27]. Extant probing birds, such as sandpipers, share long jaws and ventrally located occiputs with azhdarchids, but differ in that the margins of their rostra are parallel rather than tapering, and in that their jaws are sub-cylindrical in cross-section (Figure 7B; [110], [111]). Many probing birds possess batteries of closely packed pits on the premaxillary and dentary tips that house pressure-sensitive Herbst corpuscles (Figure 7A; [110]), but analogous features are absent in azhdarchids. Azhdarchids also lack the pleurokenetic bills of probers or the large retroarticular processes of ‘gaping’ birds [101] prompting the question as to how they could obtain infaunal prey once they had located it. The relatively small feet of azhdarchids suggest they were not adapted for supporting their weight on soft substrates where probing would be best facilitated, a point particularly pertinent for the largest forms given their potential masses of around 250 kg. The size of these forms also dictates that they would need to process enormous amounts of probed invertebrates to sustain themselves. This, in concert with the absence of cranial specialisations for probing, the relatively inflexible neck, and proportionally small feet lead us to conclude that the probing hypothesis can be rejected.

#### Mid-air predation

The possibility that azhdarchids were aerial predators of smaller flying animals [18] has not gained acceptance among pterosaur workers. Unlike modern raptorial birds, pterosaurs hawking airborne prey would have to rely on their jaws for prey capture rather than their limbs: employment of any limb in mid-air prey capture would compromise the wing membrane and stall the wing. Correspondingly, azhdarchids do not bear raptorial claws on any appendage that could be used to subdue prey in this manner. Extant volant tetrapods that employ oral apprehension of aerial prey have short, wide skulls and often possess deep mandibular symphyses [112], [113], a condition that contrasts markedly with the elongate, narrow azhdarchid skull They also tend to be relatively small, fast and agile fliers with below-average wing loading and moderately high aspect ratios [85], but while the wing loadings of modern aerial hawkers are comparable to those restored for azhdarchids, their aspect ratios are far higher. Consequently, azhdarchids would be slow, cumbersome fliers in comparison. Members of the pterosaur clade Anurognathidae conform to these criteria far better and are hence usually regarded as having been aerial insectivores convergent with swifts, nightjars and some microbats [23], [24], [114]. Given that azhdarchids were large, relatively narrow-skulled animals with stiff necks and wings better adapted for gentle gliding than high-velocity pursuit, the hypothesis that they were capable of routine aerial predation of other volant animals can be rejected.

#### Swimming and diving

Although many modern birds regularly swim or dive in pursuit of food, there is no anatomical evidence that azhdarchids did the same [contra. 16,18]. Extant birds demonstrate multiple approaches to feeding on and in water, including surface feeding, plunge diving, and surface diving [101]. Tetrapods that habitually swim possess limbs modified to greater or lesser degrees for propulsion through water, and those that regularly dive bear streamlined bodies to minimise drag [115]. With their relatively small, narrow feet, expansive wings and ventrally oriented skulls atop long, stiffened necks, azhdarchids lack both the propulsion mechanisms and streamlining for efficient movement through water and their limbs show no modifications (e.g. enlarged olecranon or cnemial processes) for swimming. Extant swimming and diving birds also hold their heads close to or above their centre of buoyancy when alighted on the water surface, but the anatomy of azhdarchid cervical vertebrae disallows the possibility of holding the neck at a high angle and may have created issues of stability if the animal were to alight on the water surface. Although other pterosaurs may have been competent swimmers [31], [116], there is no anatomical evidence that azhdarchids were suited for an aquatic existence. Rather, the elongate, slender limbs and proportionally large neck and skull of azhdarchids probably cast them as some of the least aquatically-adapted of all pterosaurs.

#### Skim-feeding

Many authors have suggested that azhdarchids were airborne piscivores [16], [18], [22], [24], [25], [27], [29], [40], and skim-feeding is often suggested as the feeding method [18], [25], [27], [30]. Both Lawson [2] and Langston [17] suggested that the lack of fish fossils and large water bodies in the *Quetzalcoatlus*-bearing Javelina Formation might refute a piscivorous diet, but the occurrence of azhdarchids in a variety of depositional settings with associated fish fossils (e.g. [58], [117], [118]) demonstrates the circumstantial nature of this argument. It has been proposed that azhdarchids used the combined lengths of their heads and necks (perhaps up to 5 m in the largest forms) to feed whilst keeping their wingtips from contacting the water, with narrow jaws minimising drag [27]. Further evidence cited for skimming includes the similarity alleged between the 50° gape of *Quetzalcoatlus* and that of *Rynchops* [25]. However, the hypothesis that azhdarchids may have been skim-feeders fails to acknowledge the remarkable and highly distinctive specialisations necessary for skim-feeding and ignores the fact that, among extant vertebrates, habitual skimming is unique to *Rynchops* [34]–[36], [115], although Royal terns *Thalasseus maximus* and Caspian terns *Hydroprogne caspia* are known to perform facultative skim-feeding behaviour [37]. The head and neck of *Rynchops* has 30 skimming adaptations [36], the most obvious being the extreme streamlining and keratinous extension of the mandibular symphysis (Figure 7C), the reinforcement and secondary bracing of the jaw joint, and the robust nature of the cervical vertebrae [35], [36]. These adaptations reflect a lifestyle that is considerably more energetically demanding and specialised than previously appreciated [75].

No azhdarchid exhibits any of these adaptations nor any functional alternatives, and in many details azhdarchids appear maladapted for skim-feeding. While slender, the dorsal surfaces of the mandibular symphyses in *Bakonydraco* and *Quetzalcoatlus* are flattened, producing triangular (rather than blade-like) cross-sections [6], [25]. Humphries et al. [75] highlighted the importance of the knife-like mandibular symphysis of skimmers in drag reduction and suggested that even small (<2 m wingspan) pterosaurs might have struggled to skim with their dorsoventrally flattened mandibles. Modelling the skimming energetics of a 10 m span azhdarchid suggests that even an unfeasibly lightweight (50 kg) individual would lack the metabolic energy to skim-feed, and this assertion applies even more to a realistically estimated 200 kg individual [75]. Also noteworthy is that, in contrast to *Rynchops*, no portion of the azhdarchid mandibular symphysis extended beyond the premaxilla, and the rhamphothecae preserved in other azhdarchoid taxa [91] suggest there was no keratinous extension either.

Crucially, other than in their gape, there is little similarity between the jaw articulation in *Quetzalcoatlus* and *Rynchops*. Although the quadrate-articular joint of *Quetzalcoatlus* shows some additional lateral bracing [25], it is fundamentally under-developed compared to the robust, doubly-reinforced jaw joint of skimmers, a morphological adaptation critical to withstanding the impacts experienced in skim-feeding [35]. Similarly, although relatively short compared to other pterosaurs, the mandibular rami of azhdarchids remain long and slender in contrast to the deep, robust rami of *Rynchops* [6], suggesting that azhdarchid jaw muscles were relatively weak compared to the enlarged, drag- and impact-resistant jaw musculature of skim-feeding birds. The necessity for these adaptations in skim-feeding cannot be understated: along with the impacts experienced on striking prey items, modern skimmers regularly strike submerged obstacles that can be severe enough to cause crashes [34], [36], [119]. Although azhdarchids could avoid these risks by trawling deeper water, modern skimmers regularly trawl shallow water bodies to maximise their chances of catching prey. We assume that azhdarchids would have had to skim in similar settings and hence would have faced similar risks: the lack of shock-absorbing structures in the azhdarchid jaw alone is compelling evidence against the skim-feeding hypothesis.

The azhdarchid neck also lacks the structures needed to cope with the drag forces and jarring impacts incurred during skim-feeding. Not only is the azhdarchid neck totally incapable of the considerable flexion needed for skim-feeding, it also lacks sculpting for strong ligament and muscle attachment that are unusually prominent in *Rynchops* [36]. The low neural spines of azhdarchid cervicals and stiffened neck structure result in very low mechanical advantage for any associated musculature. This contrasts strongly with the short neck of *Rynchops* that produces high mechanical advantage through relatively tall neural spines and pronounced dorsal curvature of the cervical series [36].

Furthermore, it is highly unlikely that ventral rotation of the azhdarchid skull at the occipital condyle could have absorbed skimming impacts in compensation for the stiffened neck. The apparent inflexibility of the axis-cervical three joint requires that all rotation took place at the occiput-axis and atlas-axis junctions, both of which are small and wholly insufficient to absorb jarring impacts. The position of the occiput also precludes extending the jaw tips to the combined length of the jaw and neck, as the skull cannot be rotated enough to align the jaws and neck on the same plane [contra. 27]. Equally, the suggested strategy of keeping the wings held at neck's length from the water surface may compromise the wing-in-ground effect benefits that are known to be important in the flight of modern skimmers [120]. Azhdarchid wings also contrast with the high aspect wings of *Rynchops* that facilitate fast, efficient ‘flap-gliding’ [36], [75], [120] rather than static soaring [contra. 30]. Hence, despite regular mention by pterosaur workers, azhdarchids lack characters that support the skim-feeding hypothesis and it is an entirely unlikely foraging method for this group of pterosaurs. Additionally, we note that the concept of skim-feeding in other pterosaurs is no more secure than it is for azhdarchids (see [75] for further details).

#### Dip-feeding

Many elements of the azhdarchid skeleton that preclude skim-feeding also apply to their inability to dip-feed (our use of the term dip-feeding here applies to the style of foraging practised by frigatebirds, gulls and terns where prey items at or near the water surface are picked up by the bird while it is on the wing). This feeding method has been proposed for azhdarchids by various authors [16], [22], [24], [29], [40], as it has for virtually all other pterosaurs. While the long necks and jaws of azhdarchids superficially resemble those of modern dip-feeders, the details of their neck and skull anatomy preclude such a foraging method. The azhdarchid neck is not flexible enough to allow the animal to reach beneath and behind the body in the manner practised by extant dip-feeders, an essential adaptation for dip-feeders given the momentum of their bodies in flight compared to their relatively stationary prey. Similarly, the ventrally-orientated occiput does not permit the azhdarchid skull to extend in line with the neck during the ‘strike’ phase of prey apprehension. Azhdarchids also lack the ventrally-curved jaw tips of many dip-feeders. Given that azhdarchids also lack the ability to apprehend prey in flight with their limbs (see discussion of mid-air predation, above), it seems highly unlikely that azhdarchids were capable of seizing prey from the water surface in flight.

#### Wading

The wading ecology proposed for azhdarchids by several workers [16], [19], [21], [31], [32] agrees with many aspects of azhdarchid anatomy and with the sedimentological context of some specimens, but has been poorly explored by its proponents. Chatterjee and Templin [16] used the occurrence of azhdarchids in lacustrine deposits as evidence of a wading lifestyle, but such argumentation is circumstantial in light of the preservation bias afforded by aquatic settings compared to terrestrial environments. Furthermore, the azhdarchid skeleton suggests that, while competent walkers, they were poor waders. The elongate limbs, neck and jaws of azhdarchids appear well suited for wading, but their manus and pes anatomy and the *Haenamichnus* footprints suggest that their extremities had relatively small surface areas, a condition quite different to the splayed feet of wading birds [101]. Some storks with relatively small feet are known to wade [109], indicating that azhdarchids may have been capable of some wading activity, but the high masses of large azhdarchids may have limited their ability to wade on soft substrates. Moreover, other pterodactyloids with larger pedal surface areas (most notably ctenochasmatoids) were almost certainly better adapted waders than azhdarchids. In view of this evidence, we suggest that azhdarchids were not habitual, although perhaps faculatative, waders.

#### Terrestrial stalkers

Our interpretation of the evidence has led us to conclude that azhdarchids severed the ties with aquatic foraging conventionally assumed for pterosaurs (e.g. [24]), and that they were instead terrestrial opportunists, finding much of their food via terrestrial, ground-level foraging. The number of bird lineages that have independently evolved to exploit such a niche suggests that it is highly plausible that some pterosaur groups could do the same. The skewed distribution of azhdarchid fossils in continental settings corroborates this hypothesis at least in part: with the possible exception of obligate scavenging, all other proposed azhdarchid ecologies would predict the occurrence of azhdarchids in other depositional contexts, but their continental preference suggests a lifestyle in which most of their time – and subsequent foraging – occurred inland. Moreover, this hypothesis most adequately explains the anatomical details that have proved problematic in other hypotheses, such as the structure and carriage of the neck and skull and relatively small feet. At the expense of swimming or wading ability, shorter feet decrease the out-lever arm of the foot during the flexion phase of the step cycle and, therefore, increase walking efficiency. Azhdarchids, therefore, possessed feet well adapted for walking: an observation that agrees with their relatively long limbs. The latter would not only increase stride length but also allow easier passage through dense vegetation and provide a high vantage point to spot prey, both adaptations reflected in modern avian terrestrial stalkers [104]. The robust foot skeleton and padded soles present further adaptations to a primarily-grounded lifestyle, providing both traction and cushioning when walking on hard substrates. An efficient standing and walking ability in azhdarchids is further verified by the unusually narrow-gauge *Haenamichnus* trackway: bringing the limbs closer to the midline allowed grounded azhdarchids to support their weight largely through compressive forces acting on sub-vertical limbs. Other trackways show that non-azhdarchid pterosaurs had partially abducted limbs when moving terrestrially [e.g. 96] and consequently would have had to exert some effort to counter bending forces acting on their limb bones and joints. That azhdarchids appear to have overcome this problem suggests that they had one of the most energy-efficient postures and gaits known in any pterosaur, and we speculate that strong specialisation to weight-bearing may have facilitated the evolution of exceptional size in the clade. Combined with their wings well adapted for flight around inland settings (see above), azhdarchids appear well suited for locomotion in cluttered terrestrial environments.

**Figure 8:**
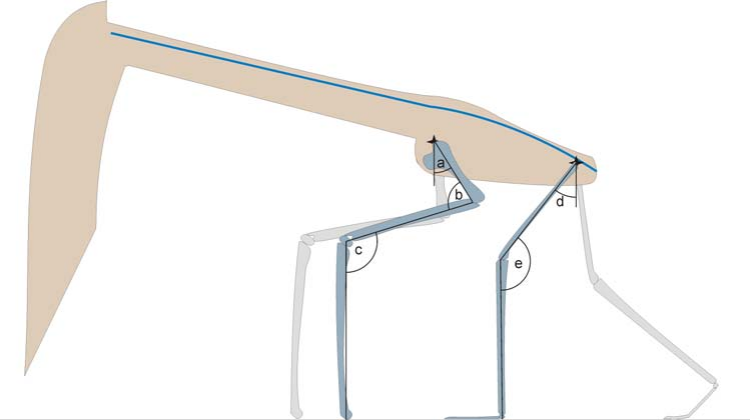
Reconstructed feeding posture of an azhdarchid with sagittally aligned limbs, as evidenced by [46]. The blue line indicates the dorsal and cervical column; note how the long jaws require little flexion of the forelimb to be lowered to the ground and how only moderate flexion of the anterior cervical series would lower the jaws fully. Letters denote approximate angles used in this reconstruction; a, 30°; b, 80°; c, 120°; d; 35°; e, 145°.

The unusual pterosaur manual morphology and lack of large claws suggests that azhdarchids did not employ their limbs in apprehension of prey, even when grounded. It is assumed, therefore, that the jaws were the primary agents of prey apprehension, and would need to be lowered to ground level to procure food. Due to the long azhdarchid hindlimb, relatively little flexion is required in the forelimbs to bring the skull and neck towards the ground (Figure 8). Moreover, the relatively simple neck and skull mobility required in terrestrial foraging means that, unlike most other purported feeding styles, the stiff azhdarchid neck and ventrally oriented occiput do not present mechanical problems for this feeding method, as only slight flexion of the anterior neck vertebrae will fully lower the jaws to the substrate. The perpendicular orientation of the skull to the neck decreases the cervical flexion required to lower the jaws and, augmented by the great length of the skull, the distance between the jaw tips and the ground. The elongate neck also serves to decrease the flexion necessary to lower the jaws, as relatively minor inter-vertebral rotations are exaggerated along the length of the series, although this in itself does not necessarily preclude the influence of other factors (e.g. counterbalance of long limbs, greater visual acuity gained by increased skull height, sexual selection) on the evolution of the azhdarchid neck. The limited mobility of the neck means azhdarchids could not, however, projectile-feed in the manner of many long-necked birds.

The azhdarchid skull has a similar construction to modern birds that habitually stalk terrestrial environments (such as marabou storks and ground hornbills; Figure 7D) in bearing a long but relatively deep rostrum that extends anteriorly without invasion of the nasoantorbital fenestra (as is typical of other azhdarchoids [e.g. 11], [12]). The long jaw and relatively small jaw muscles of azhdarchids would presumably limit them to small food items that would not require strong bite forces or high mechanical strength to subdue or process. As with extant avian terrestrial stalkers, the generalised bills of azhdarchids would enable them to have a broad carnivorous diet comprised of relatively small vertebrates and large invertebrates, possibly supplemented with fruit [6] and carrion (see above).

**Figure 9:**
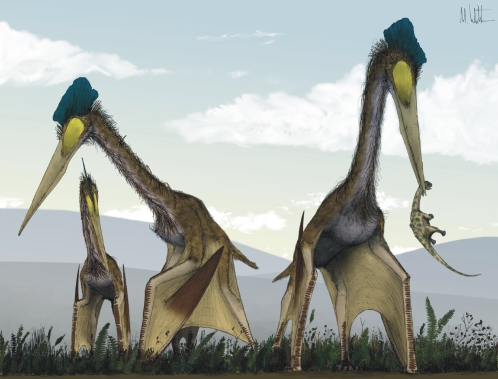
Life restoration of a group of giant azhdarchids, *Quetzalcoatlus northropi*, foraging on a Cretaceous fern prairie. A juvenile titanosaur has been procured by one pterosaur, while the others stalk through the scrub in search of small vertebrates and other foodstuffs.

Hence, although azhdarchid anatomy is unique in a number of aspects, they appear to have been stork- or ground hornbill-like terrestrial stalkers (Figure 9), with the best modern analogues being the most generalized storks, such as the *Ciconia* species. Note that azhdarchids lack the specializations seen in some stork taxa, such as the *Mycteria* wood storks (which specialise in tactile feeding and wading), or *Anastomus*, the open-billed stork (which possesses scopate tomial edges and upper and lower jaws that bow away from each other. These are apparently specializations that assist in the holding of hard-shelled prey [109]).

### Concluding remarks

Studies of pterosaur ecology have suffered from the dogmatic attitude that pterosaurs were predominately aerial piscivores living in coastal settings, in spite of steady accretion of evidence that they occupied a variety of ecological roles in a suite of environments. The unusual anatomy of azhdarchids strongly indicates that they had a unique ecology and inhabited unusual environments compared to many other pterosaurs: these details have been overlooked by most authors who have interpreted azhdarchids as marine piscivores occupying niches conventionally considered typical of pterosaurs as a whole. This unusual lifestyle may explain the resilience of azhdarchids to decline in contrast to other Cretaceous pterosaur lineages, few or none of which persisted to the late Maastrichtian as did azhdarchids. It is hoped that this re-revaluation of azhdarchid ecology will inspire much-needed descriptions of azhdarchid material, empirical testing of the hypotheses presented here, and further research into the lifestyles of pterosaurs beyond their flight capability.
